# GaitKeeper: An AI-Enabled Mobile Technology to Standardize and Measure Gait Speed

**DOI:** 10.3390/s24175550

**Published:** 2024-08-28

**Authors:** Naomi Davey, Gillian Harte, Aidan Boran, Paul Mc Elwaine, Seán P. Kennelly

**Affiliations:** 1 Institute of Memory and Cognition, Tallaght University Hospital, D24 NR0A Dublin, Ireland; gillian.harte@tuh.ie (G.H.); sean.kennelly@tuh.ie (S.P.K.); 2 Department of Medical Gerontology, School of Medicine, Trinity College Dublin, 2 Dublin, Ireland; 3 Department of Physiotherapy, Tallaght University Hospital, D24 NR0A Dublin, Ireland; 4 Insight Centre, Dublin City University, Collins Ave Ext, Whitehall, 9 Dublin, Ireland; 5 Digital Gait Labs, Glasnevin, 9 Dublin, Ireland

**Keywords:** gait analysis, mobile health technology, artificial intelligence, gait speed measurement, augmented reality, clinical assessment tools, health indicators, movement analysis techniques, healthcare innovation

## Abstract

Gait speed is increasingly recognized as an important health indicator. However, gait analysis in clinical settings often encounters inconsistencies due to methodological variability and resource constraints. To address these challenges, GaitKeeper uses artificial intelligence (AI) and augmented reality (AR) to standardize gait speed assessments. In laboratory conditions, GaitKeeper demonstrates close alignment with the Vicon system and, in clinical environments, it strongly correlates with the Gaitrite system. The integration of a cloud-based processing platform and robust data security positions GaitKeeper as an accurate, cost-effective, and user-friendly tool for gait assessment in diverse clinical settings.

## 1. Introduction

In recent years, gait speed has emerged as a valuable health indicator, particularly among older adults. Its strong correlations with all-cause mortality, functional status, general health, mobility, disability, and rehabilitation effectiveness underscore its ability to predict health outcomes [1,2,3,4,5]. Reflecting its growing recognition as an important health metric, gait speed was dubbed the ‘sixth vital sign’ in Fritz and Lusardi’s White Paper [6]. This paper introduces GaitKeeper, an innovative artificial intelligence (AI)-enabled mobile application designed to standardize the measurement of gait speed across diverse clinical settings, thereby enhancing the consistency, operational efficiency, and accessibility of gait speed assessments.

Gait speed is not just a simple health metric; it is a powerful predictive tool, effective in identifying conditions like frailty. Frailty is characterized by reduced physical and functional reserves, thereby increasing an individual’s vulnerability to various stressors [7]. Slow gait speeds are linked to heightened levels of frailty and pre-frailty, underscoring its predictive capacity for health outcomes including early mortality, severe disability, increased fall risk, and the need for hospitalization [8,9].

Additionally, reductions in gait speed have been associated with cognitive decline. Specifically, the transition from mild cognitive impairment (MCI) to dementia has been linked with reductions in gait speed over time [10,11]. In cardiovascular health, gait speed had been shown to identify patients at an increased risk of cardiac incidents, providing prognostic information for managing conditions such as heart failure in older adults [12,13]. Its proven predictive accuracy for mortality within 30 days to one year post-cardiac surgery highlights its value in surgical risk assessments among older adults [14,15,16].

Furthermore, gait speed assessment is an important part of fall risk management. It is estimated that 30% of adults over 65 years of age experience falls annually, significantly impacting morbidity and mortality [17,18]. According to the World Guidelines for Falls Prevention and Management, a usual pace gait speed over a 4 m distance is the most robust predictor of falls [19,20]. Integrating regular gait speed assessments within clinical protocols can facilitate timely and potentially more effective interventions.

Despite its utility, the practical implementation of gait speed measurement faces significant challenges due to the lack of standardization and variable accessibility of gait assessment tools. Methodological inconsistencies, such as variations in walkway length, starting conditions, and the choice of timing mechanisms can result in variable measurements of gait speed.

Walkway length is important when measuring gait speed. Longer walkways capture a more accurate representation of an individual’s natural gait pattern, including steady-state walking speed. Although less physically demanding, shorter walkways might not capture the complete range of a person’s gait, potentially leading to underestimation or overestimation of their true speed [21]. However, longer walkways can be tiring, especially for those with mobility impairments, impacting their walking speed and endurance which could compromise the accuracy of the assessment. Therefore, it is essential to balance the walkway length with the individual’s physical capabilities to ensure precise and reliable gait analysis.

Disparities in how subjects begin or end their walk due to inconsistent acceleration or deceleration phases, coupled with differing instructions given to subjects, can further impact the accuracy of these measurements [22]. The choice of timing mechanisms, such as manual stopwatches versus automated sensors, also introduces variability in measurement precision and consistency. Clear, consistent directives are important for reliable and replicable measurements. These constraints often cause clinicians to rely on observational analysis, which undermines the precision and reliability of gait assessments, thus compromising gait speed’s effectiveness as a clinical tool [23].

Recognizing the limitations of traditional gait speed measurement methods, researchers have explored advanced techniques such as instrumented gait analysis using wearable sensors and pressure mat systems [24]. These technologies offer enhanced precision and objectivity in capturing detailed gait data. However, their adoption in clinical practice is often restricted by high costs, spatial requirements, and the need for specialized training [25,26]. Establishing standardized assessment methods is crucial to fully harness the insights that gait speed can offer about a patient’s health and functional capabilities.

The landscape of gait analysis has been transformed by advancements in AI and machine learning. Traditionally, convolutional neural networks (CNN) and long short-term memory networks (LSTM) have been used separately to analyze gait patterns, but often suffered from loss of time-series and spatial information due to inherent limitations [27,28]. The Sequential Convolution LSTM Network (SConvLSTM) combines the strengths of both CNNs and LSTMs to capture the more nuanced spatial and temporal dynamics of gait [29]. This integration has improved the assessment of complex gait patterns, particularly in crowded or dynamic environments, marking an improvement over earlier technologies that used CNNs or LSTMs separately.

Furthermore, Radial Basis Function (RBF) networks offer improvements in handling the non-linear aspects of gait assessment. RBF networks, primarily used for function approximation tasks are beneficial in scenarios where the relationship between input variables and expected output is complex and cannot be easily modeled with traditional linear predictors. Feng et al. integrated dynamic RBF networks with an error-based feature fusion strategy to improve the analysis of frontal-view gait sequences [30]. This strategy, employing the pre-trained Inception-ResNet-v2 model, has improved feature extraction whilst also adapting to changes in gait driven by varying walking speeds or environmental factors.

Moreover, the Dynamic Aggregation Network (DANet) enhances the analysis of temporal data sequences by combining dynamic attention mechanisms with self-attention processes [31]. DANet adapts to variability in a patient’s gait caused by external factors such as clothing or carrying conditions, ensuring high precision in gait detection across diverse environmental settings. This adaptability is valuable for continuous monitoring and assessment in both clinical and research settings.

The ongoing development of deep learning methods and sophisticated neural network architectures continues to significantly advance gait measurement tools such as GaitKeeper. The integration of such advanced technologies in gait analysis not only facilitates better health outcomes but also sets new standards for the accuracy and applicability of gait speed measurements in real world settings.

## 2. Materials and Methods

**Figure 1:**
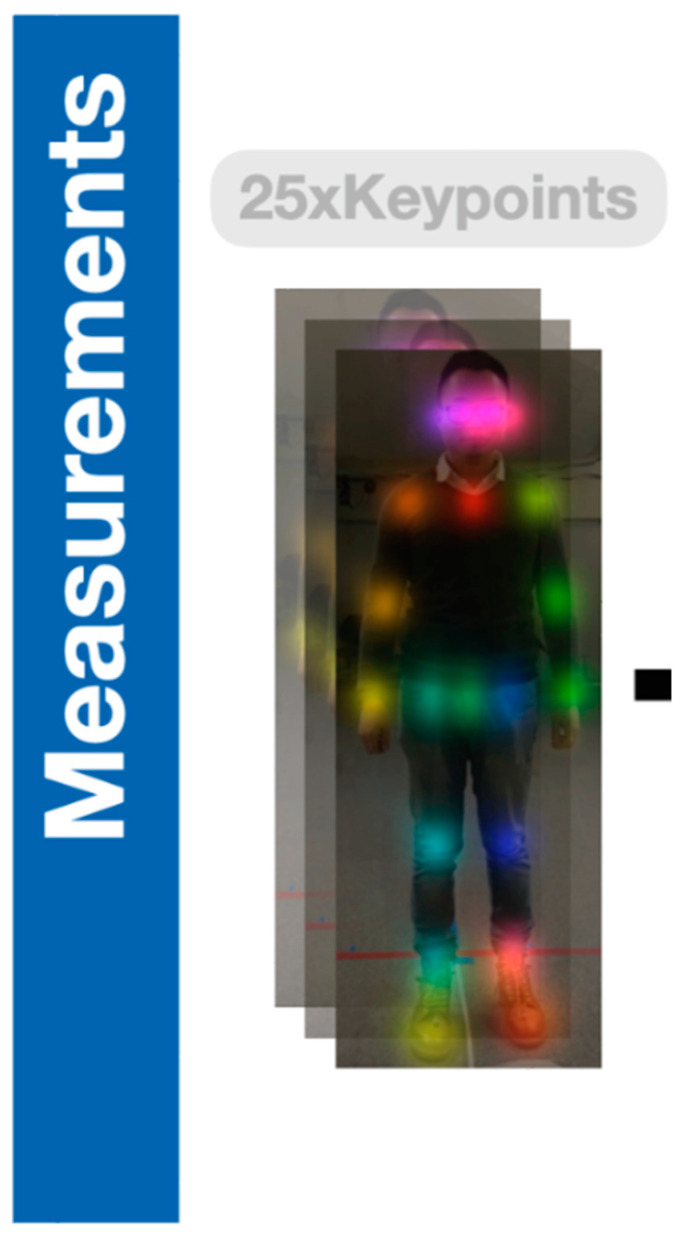
Twenty-five joint positions across the body are captured by GaitKeeper for each video frame.

GaitKeeper uses AI to manage and process human gait data. The AI-driven engine is designed to identify 25 distinct joint positions from the recorded videos (Figure 1). GaitKeeper processes these at a rate of 60 frames per second for detailed analysis of gait dynamics. This high-frequency joint analysis helps in understanding subtle deviations in gait.

**Figure 2:**
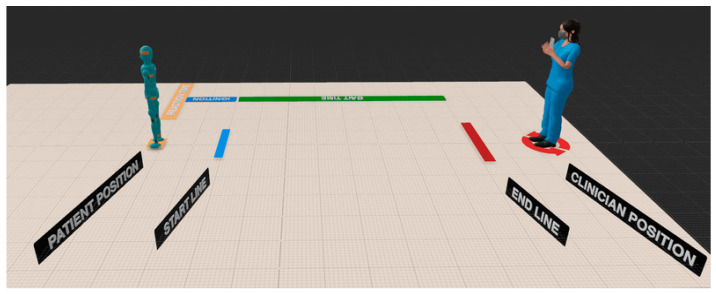
GaitKeeper’s AR Gait Lab Virtual Walkway: This illustration shows a clinician with a smartphone, stationed beyond the end line, over four meters away from the patient. The patient is positioned in front of the start line, prepared to commence the walking assessment.

**Figure 3:**
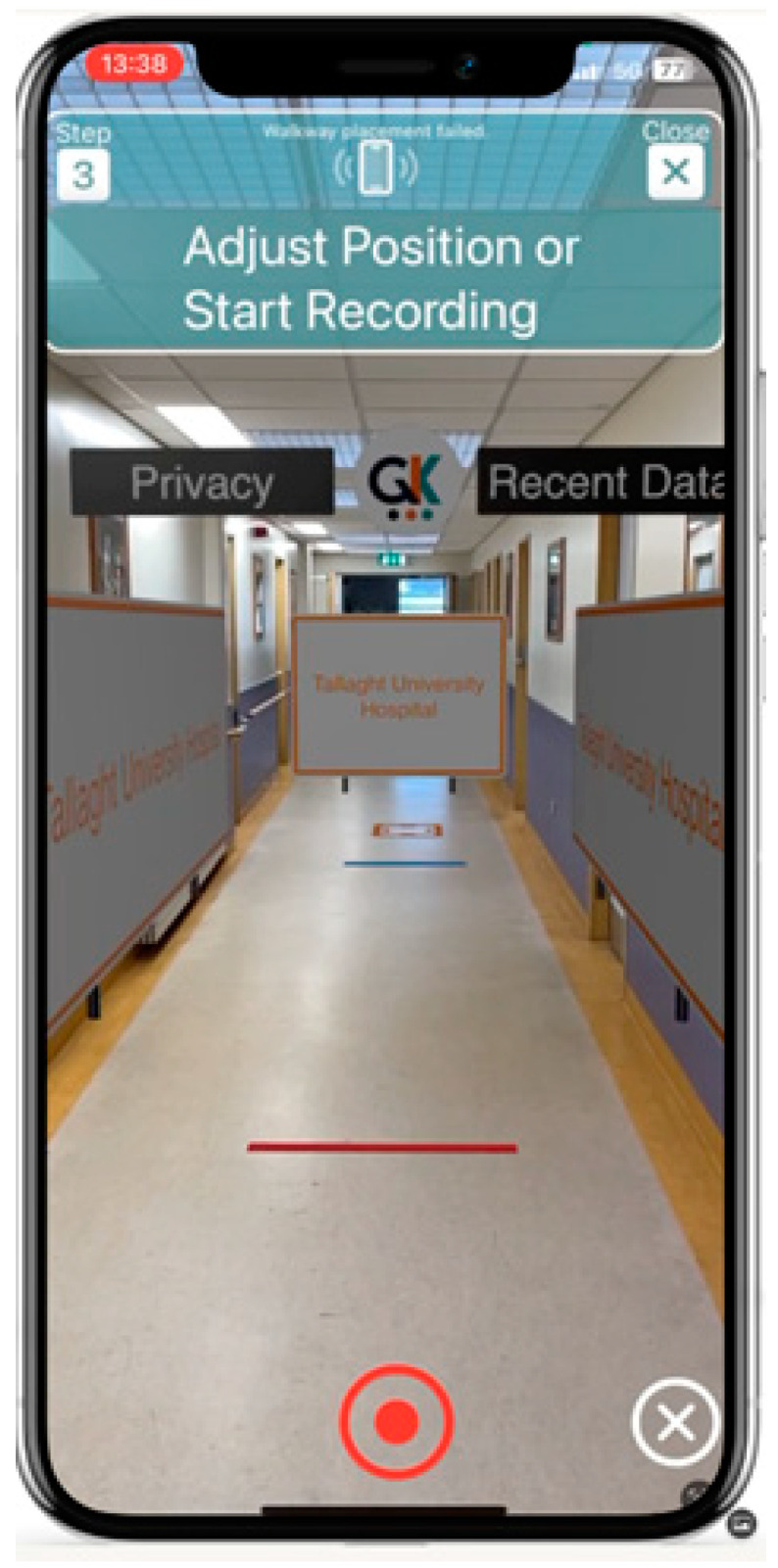
Clinician’s View of the AR Gait Lab: This figure shows the clinician’s perspective through a smartphone including the virtual walkway ready for use, moments before initiating the walking assessment in the AR environment.

GaitKeeper creates a virtual gait laboratory, employing augmented reality (AR) to simulate a controlled environment that surpasses the traditional gait lab settings in consistency and adaptability. This virtual environment includes a detailed walkway complete with starting and ending lines, directional signs, interactive feedback, and privacy features to simulate a gait analysis laboratory (Figure 2 and Figure 3).

By integrating AI and AR, GaitKeeper enhances video data analysis, capturing spatiotemporal metrics for precise gait analysis. This automated process records moments of the gait assessment process, such as the initiation and completion of assessments, as well as reaction and ignition times.

### 2.1. Pipeline Overview

**Figure 4:**
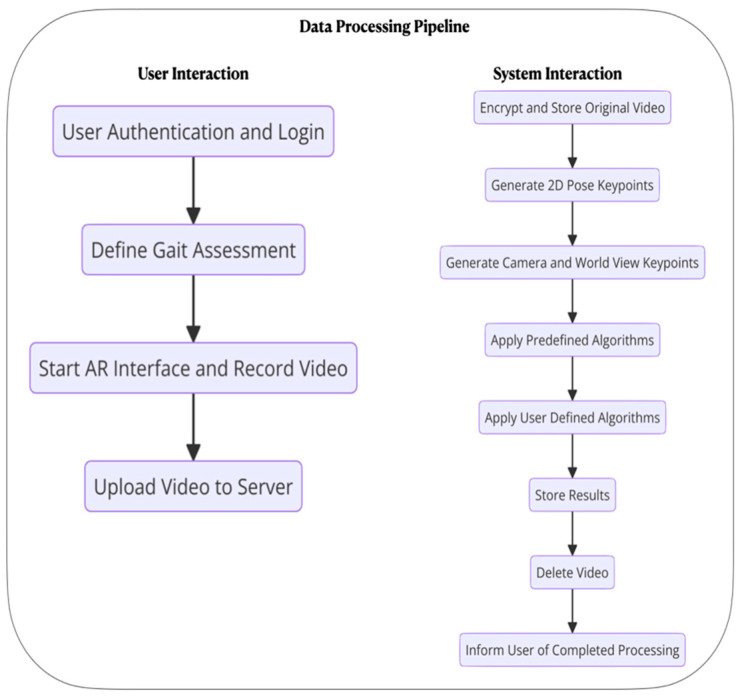
Data processing pipeline. An overview of the processing pipeline from video recording to gait data including the coordinate systems computed and key (but not exhaustive) processing modules.

The GaitKeeper data processing pipeline starts with user authentication and login via the GaitKeeper app (Figure 4). Following login, users define a gait assessment by inputting the subject’s identifier, and confirming consent. The app’s AR interface guides the user to record a gait assessment, with standardized virtual markers marking the start and ending points of the test. Once the recording is complete, the video is securely uploaded to the GaitKeeper server through an application programming interface (API).

Upon upload, the video undergoes encryption for security and is then stored on the GaitKeeper servers. The processing phase begins with the generation of 2D pose keypoints, adjusted according to the current system configuration. This is followed by the creation of camera and world view keypoints. Predefined algorithms are used to analyze the data, supported by user-defined algorithms to tailor the analysis to specific assessment needs. Once analysis is complete, the results are stored, and the original video is deleted to maintain privacy. Finally, the user is notified of the completion of processing.

Beyond data collection, the GaitKeeper server performs calculations to determine various gait parameters including gait speed, stride length, and step characteristics. GaitKeeper calculates detailed positional angles at key joint areas—hip, knee, and ankle—as well as the base of support and foot strike events, creating a comprehensive overview of body segment motion during walking, referred to as ‘kinematics’. Understanding gait kinematics can help to pinpoint gait abnormalities, improve performance and identify targets for rehabilitation [32].

### 2.2. Coordinate Systems Overview

GaitKeeper employs three distinct coordinate systems to accurately model and analyze motion captured in gait analysis.

1. Screen View: The screen view provides a 2D representation of keypoints identified on the subject’s body, captured in video format. Each incoming key point format is translated into a standardized format (joint, x, y, quality, timestamp) as shown in Equation (1) below. A series of such points is represented in S.

**Equation (1).** Screen view format.


EQ1: (xi, t, yi, t, qi, t, t) for t ∈ t0, t1, …, tn
S = (Ji, xi, t, yi, t, qi, t, t)∣i = 1, 2, …, 25 and t ∈ t0, t1, …, tn
(1)

2. Camera View: The camera view enhances the keypoint data by incorporating camera-specific parameters, enriching the data set with perspective corrections for precise spatial analyses. Denoting f_x_ and f_y_ for the focal lengths in the x and y directions, respectively, and c_x_ and c_y_ for the principal point offsets in the x and y directions, respectively. Coordinates are adjusted based on camera settings to provide a consistent data set across different recording devices. The normalized x and y coordinates are shown in Equations (2A) and (2B) and a series of such points in S (Equation (2)).

**Equation (2).** Camera view coordinates.

(2A) $$x_{i,t}^{’}=\frac{x_{i,t}-c_{x}}{f_{x}}\;\;\;(A)$$

(2B) $$y_{i,t}^{’}=\frac{y_{i,t}-c_{y}}{f_{y}}\;\;\;(B)$$

where $S=\left\{\left(J_{i},x_{i,t}^{’},y_{i,t}^{’},q_{i,t},t,f_{x},f_{y},c_{x},c_{y}\right)|i=1,2,\ldots ,25\;and\;t\in \left\{t_{0},t_{1},..t_{n}\right\}\right\}.$

3. World View: The world view is a 3D representation that integrates depth data collected via augmented reality markers for the start position, end position and 10 cm waypoints. This is used for applications requiring spatial depth analysis, such as assessing the 3D movement patterns of different body segments during a gait cycle (Equation (3)).

**Equation (3).** World view data points.

(3) $$S=\left\{\left(J_{i},\frac{x_{i,t}-c_{x}}{f_{x}}.\frac{z_{0}}{z_{i,t}},\frac{y_{i,t}-c_{y}}{f_{y}}.\frac{z_{0}}{z_{i,t}},\frac{z_{i,t}}{z_{0}},q_{i,t},t\right)|i=1,2,\ldots ,25\;and\;t\;\in \left\{t_{0},t_{1},\ldots t_{n}\right\}\right\}.$$

GaitKeeper introduces a ‘digital gait lab’, for collecting human motion data (Figure 2 and Figure 3). The digital gait lab combines three types of data—2D key point data, camera data, and augmented reality depth information—collected during video recording. It uses three coordinate systems to generate motion views: the screen view, screen view adjusted for camera parameters, and a world view created by integrating depth data from augmented reality.

A suite of apps (APP), API and data processing modules (DPM) allow videos to be recorded using a standard mobile phone camera, securely uploaded to the cloud and processed using a range of administrative and kinematic algorithms. The goal of the platform is to simplify the collection of objective and standardized human motion metrics in a clinical setting.

### 2.3. Data Processing Modules Overview

Using these three views of motion, the processing modules apply kinematic functions to the keypoint dataset to extract meaningful gait metrics:

1. Signal Smoothing: Keypoint data can be smoothed as required by user defined modules or system defined modules, using a custom Savitzky–Golay filter that accounts for the subject’s distance from the camera. The smoothed value at point i (ŷ_i_), considering the adjusted window size and polynomial degree, is shown in Equation (4).

**Equation (4).** Signal smoothing formula.

(4) $$\left[\hat{y_{i}}=\sum_{j=-m_{i}}^{m_{i}}c_{j}\left(d_{i}\right)y_{i+j}\right]$$

The coefficients c_j_ (d_i_), are calculated using polynomial fitting within the window size m_i_ and polynomial degree d_i_.

2. Joint angle measurement: Joint angle measurements are calculated using three points (a, b and c), where point b is the vertex and points a and c form the angle with point b. The angle between vectors ab and ac is calculated as outlined in Equation (5).

**Equation (5).** Joint angle calculation formula.

(5) $$\theta =\cos^{-1}\left({\left(x_{a}-x_{b}\right)}^{2}+{\left(y_{a}-y_{b}\right)}^{2}{\left(x_{c}-x_{b}\right)}^{2}+{\left(y_{c}-y_{b}\right)}^{2}\left(x_{a}-x_{b}\right)\left(x_{c}-x_{b}\right)+(y_{a}-y_{b})(y_{c}-y_{b})\right).$$

3. Gait Event Detection: The system supports the detection of a range of gait events, including Heel Strike, Toe Off, Heel Rise, Feet Adjacent, Tibia Vertical, and the Stance and Swing Phases. For instance, Toe Off events are determined by identifying the frames corresponding to the troughs in the gait cycle data. Troughs are defined as consistent lowest local mean of a foot related keypoint (e.g., ankle or toe).

Due to patent restrictions, further details about the CNN architecture within the Gaitkeeper system are proprietary. However, the advanced deep learning techniques involved are pivotal in delivering fast and accurate analyses.

**Figure 5:**
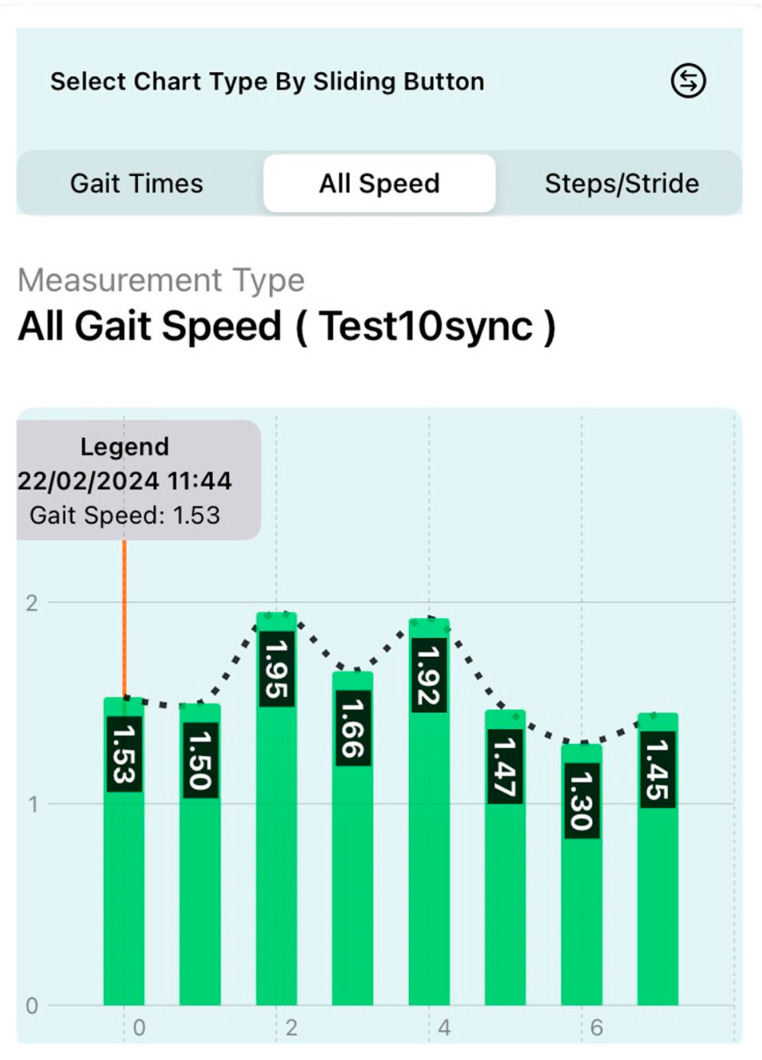
Results view of longitudinal monitoring, displaying distinct gait speed measurements taken at eight separate time points.

Typically, gait test results are available within three minutes, providing timely insights into gait mechanics. The system’s ability to generate visually detailed charts enables comprehensive longitudinal monitoring, enhancing clinical decision-making based on dynamic patient data, as demonstrated in Figure 5.

GaitKeeper employs stringent data security protocols to safeguard the integrity and confidentiality of sensitive data. The system uses the computational power of Graphical Processing Units (GPUs) like the Nvidia Tesla V100, which processes video data through a 32-layer convolutional neural network. Using Microsoft Azure Cloud, GaitKeeper can use up to four GPU’s simultaneously, facilitating efficient workload management and rapid data processing.

To secure data during transmission, GaitKeeper employs Transport Layer Security (TLS) encryption to reduce the risk of interception or manipulation. The system enforces strict access controls to regulate user interactions, network communication, and device connectivity, ensuring that only authorized personnel can access the system. Moreover, GaitKeeper protects data at rest with an encrypted NoSQL database to prevent unauthorized access.

Active monitoring of system and user activities is done to quickly detect and respond to any irregular or unauthorized activities. GaitKeeper also prioritizes user privacy by anonymizing video recordings, specifically through the removal of facial features, to maintain individual anonymity across data sets.

### 2.4. Clinical Validation of Gaitkeeper

This project involved two phases of testing to evaluate GaitKeeper. In both phases, participant’s gait speed, steps and stride length were recorded. All participants mobilized independently, wearing their own clothes and shoes, without the use of any aids.

Phase One involved comparing GaitKeeper with the Vicon system, a 3D motion capture technology regarded as the gold standard in gait analysis [33]. This phase involved 35 healthy volunteers from Dublin City University, aged 20 to 32 years, with equal gender distribution—50% female. Participants were asked to complete a series of five-meter walk tests, measured contemporaneously by Vicon and GaitKeeper. Participants had five Vicon markers attached to their feet, neck (rear), and sternum. They completed multiple five-meter walking tasks at slow, normal, and fast paces, including under dual-task conditions. Each test scenario was repeated three times, resulting in a total of nine recordings per participant.

Phase Two took place at a memory clinic in a large teaching hospital, involving 30 participants over 55 years of age, all diagnosed with mild cognitive impairment. This phase involved a comparison of GaitKeeper with the GAITRite system, another benchmark gait analysis tool that features a pressure-activated sensor walkway, that measures spatiotemporal variables of gait [34,35]. Participants completed eight four-meter walking tasks—two at their usual pace, two at a fast pace, two while engaged in a cognitive dual task, and two during a motor dual task. Each walk was concurrently measured by both GAITRite and GaitKeeper.

## 3. Results

**Figure 6:**
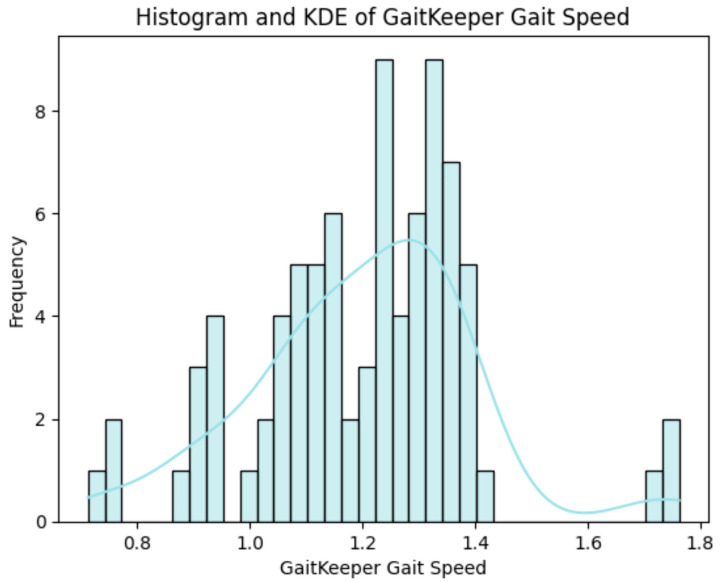
Phase One: Variation in gait speed across tests.

**Figure 7:**
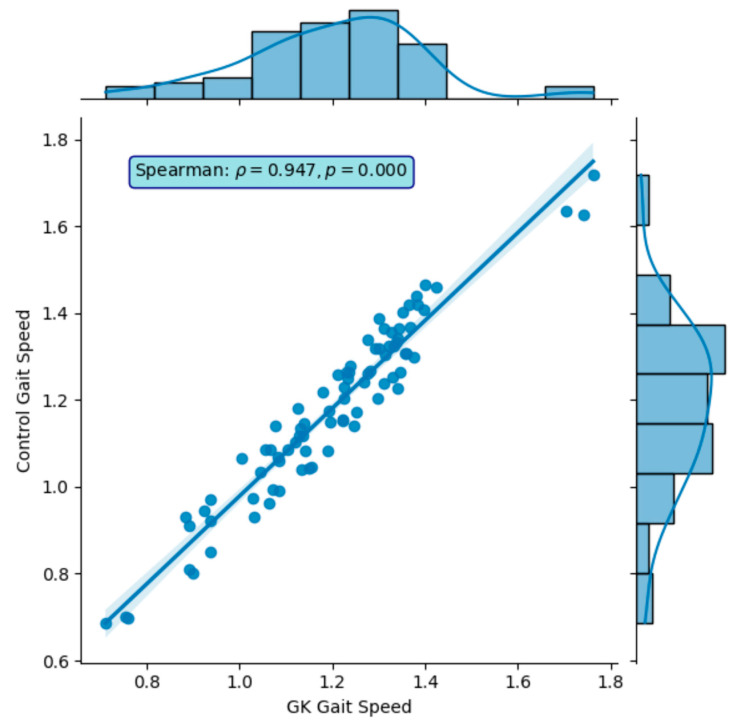
Phase One: Correlation between GaitKeeper and Vicon (gait speed)—external consistency: Spearman coefficient 0.947 (*p* < 0.0001).

**Figure 8:**
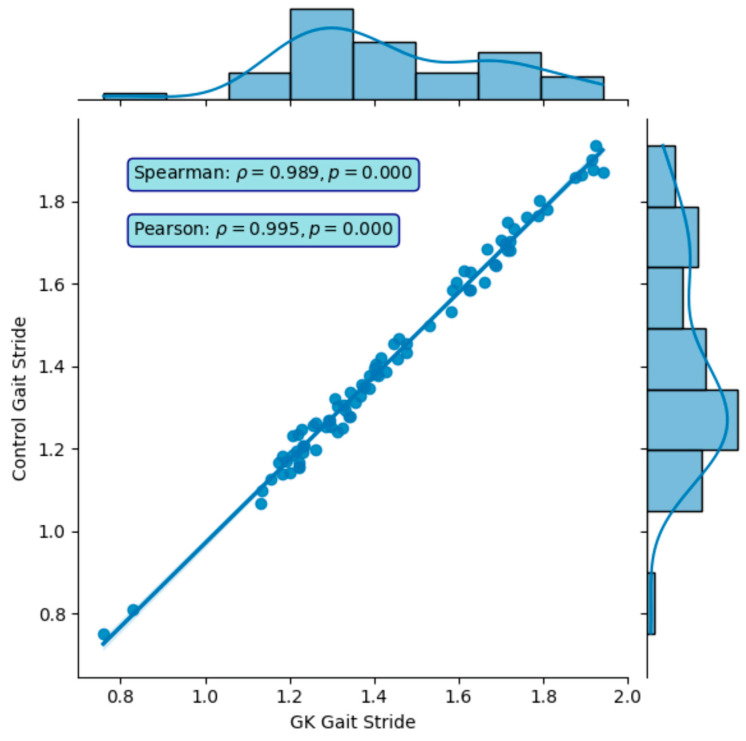
Phase One: Correlation between GaitKeeper and Vicon (stride length)—external consistency: Spearman coefficient 0.989 (*p* < 0.0001).

In Phase One, nine participants completed a total of 81 gait tests. The gait profiles exhibited speeds ranging from of 0.8 to 1.2 m/s, stride lengths between 65 to 80 cm, and a body swing of less than 1 m (Figure 6). The results demonstrated that GaitKeeper’s measurements of gait speed, stride length, and step length were within 2% variance compared to the Vicon system. High correlation values were evident, with Spearman coefficients of 0.947 for gait speed and 0.989 for stride length, both highly significant (*p* < 0.0001) as demonstrated in Figure 7 and Figure 8.

**Figure 9:**
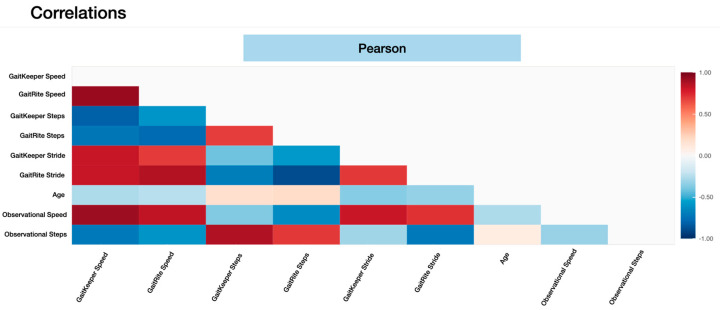
Phase two: Correlation between GaitKeeper and GaitRite for multiple gait parameters. External consistency: Pearson correlation coefficient (*p* < 0.0001 all tests).

**Figure 10:**
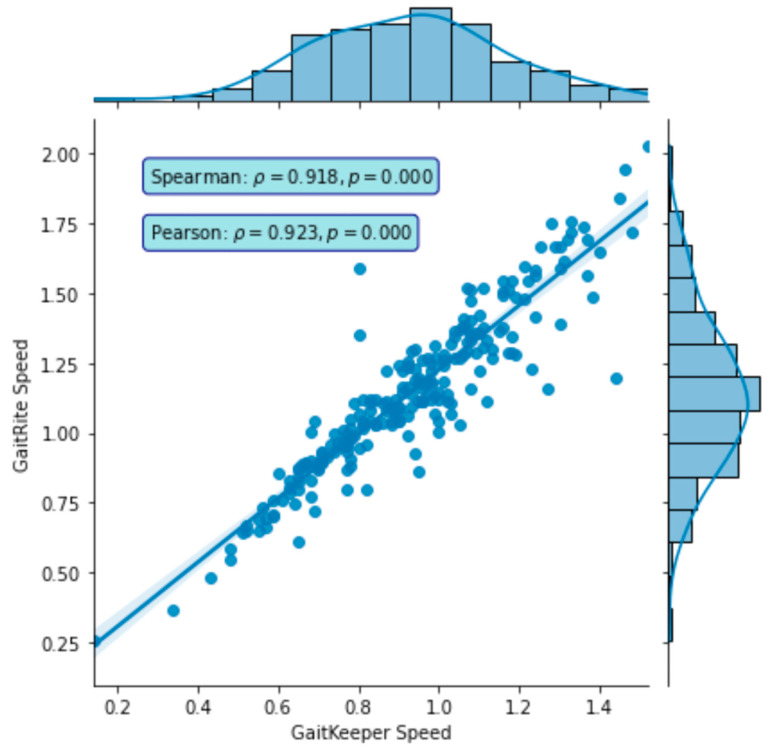
Phase Two: Correlation between GaitKeeper and Vicon (gait speed)—external consistency: Spearman correlation coefficient (*p* < 0.0001).

**Figure 11:**
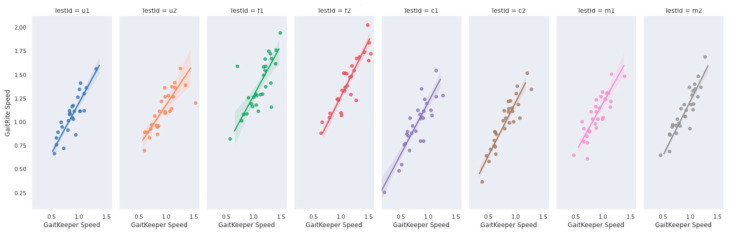
Phase two: correlation between GaitKeeper and Vicon (gait speed)—internal consistency: Pearson correlation coefficient (*p* < 0.0001 all tests).

Phase Two of the study concluded with a total of 240 gait tests. The data revealed a strong correlation between measurements from Gaitrite and GaitKeeper. Pearson correlation coefficients for steps and stride length were 0.72 and 0.71, respectively, both highly significant (*p* < 0.0001). Additionally, the Spearman correlation coefficient for gait speed was 0.918 (*p* = 0.000) indicating a high degree of consistency between the two systems in capturing gait speed dynamics (Figure 9 and Figure 10). Furthermore, internal consistency was confirmed with robust Pearson correlation coefficients across all tests (*p* < 0.0001), further substantiating the reliability of GaitKeeper (Figure 11).

## 4. Discussion

GaitKeeper revolutionizes traditional gait analysis, which often relies on cumbersome setups such as multi-camera systems or simple observational methods. By simplifying the process to a single video recording on devices like smartphones or tablets, GaitKeeper offers a user-friendly and efficient alternative.

The use of a four-meter digital gait lab in GaitKeeper standardizes testing conditions while excluding initial acceleration, ensuring consistency and reliability in clinical assessments. Research has demonstrated that a usual pace gait speed measured over this distance is a robust predictor of fall risk, with speeds below 0.8 m per second significantly increasing the likelihood of falls among older adults [20].

GaitKeeper shows strong correlations with established systems like Vicon (Spearman coefficient of 0.947) and GaitRite (0.918) for gait speed, and with Vicon for stride length (Pearson coefficient of 0.995) and GaitRite for step count (0.71). These correlations validate GaitKeeper as a robust tool for clinical assessments.

The innovation of GaitKeeper lies in its ability to create a realistic and standardized virtual gait analysis environment, thereby reducing variability in traditional assessments. Leveraging AR for a realistic virtual gait lab setting and AI for precise, real-time analysis, Gaitkeeper provides accessible and accurate measurements of gait speed.

As a Class 1 medical device with a Conformité Européenne (CE) mark, GaitKeeper significantly advances gait analysis. It is particularly useful in gerontological care, rehabilitation, and preventive medicine, where gait speed is a valuable health indicator. The mobile application sets a new standard in gait analysis, enhancing clinical insights and supporting a range of diagnostic and therapeutic decisions. The capability for longitudinal monitoring is particularly beneficial for managing chronic conditions and rehabilitation programs, allowing clinicians to detect subtle changes over time, such as deteriorations in health or response to rehabilitation programs. GaitKeeper’s accurate data collection also supports healthcare research by contributing to the understanding of gait dynamics and aiding in the development of targeted interventions.

GaitKeeper has the potential to enhance patient engagement by monitoring rehabilitation progress, encouraging adherence to therapeutic programs, and instilling a sense of ownership over health outcomes. Its portability and utility allow for use in non-traditional settings such as patient homes, outpatient clinics, and telehealth initiatives, facilitating remote monitoring of gait and early detection of mobility issues. This adaptability is particularly beneficial in expanding the accessibility of advanced gait analysis and integrating it into virtual care models. Additionally, GaitKeeper’s can detect subtle nuances in gait, signaling early changes that may indicate emerging health concerns.

Expanding beyond gerontological applications, GaitKeeper has the potential to improve diagnostics and patient care across the healthcare spectrum. For neurological conditions such as Parkinson’s disease, multiple sclerosis, and Amyotrophic Lateral Sclerosis, GaitKeeper’s ability to track subtle gait changes could provide evidence of disease progression or changes in treatment responsiveness. In pediatric care, it could be used to monitor gait patterns to detect and track developmental motor delays, contributing to timely interventions and improved monitoring of motor milestones. For preoperative evaluations, precise measurements of gait speed could serve as a screening tool to identify candidates for prehabilitation programs, helping to optimize outcomes and positively influence the post-operative course. Furthermore, in sports medicine, GaitKeeper can assess gait speed and mechanics in athletes, facilitating performance adjustments through biomechanical analysis.

This broad applicability underscores GaitKeeper’s role in innovative patient care by providing reliable data for various therapeutic and diagnostic applications.

## 5. Conclusions

The findings of this study confirm GaitKeeper as a reliable and precise tool for gait analysis, showing significant concordance with the established GaitRite system. GaitKeeper’s accuracy in measuring gait speed, steps, and stride length, combined with its user-friendly and cost-effective features, position it as an ideal solution for both clinical and research applications. The integration of AI and AR not only enhances the accuracy and consistency of gait speed assessments but also sets a new benchmark in gait analysis technology.

GaitKeeper’s adaptability will increase the availability and ease of gait speed assessments, allowing integration into diverse care models. As global populations age, healthcare systems must adapt to assess older adults in a variety of settings. Recognizing that not all patients are seen in hospital settings, it is important to have adaptable tools that can reliably detect subtle signs of clinical deterioration in both clinical and non-clinical environments. Using gait speed as a ‘sixth vital sign’ for assessment and triage allows clinicians to perform gait analyses without bulky equipment or specialized facilities.

Future research will focus on incorporating patient feedback to tailor the device’s functionality, thereby enhancing its effectiveness across varied settings. These efforts will aim to provide personalized care, enabling targeted and effective interventions based on individual gait patterns and specific health needs.

While acknowledging the limitations of this focused, single-center cohort study, further research across multiple locations is necessary to confirm these results. Such expansion will enhance the generalizability of these findings and allow for adjustments to different cultural and logistical environments, ensuring that GaitKeeper meets a broad range of global healthcare needs.

GaitKeeper represents a major advancement in gait analysis technology, with the potential to standardize assessments and improve patient outcomes. As healthcare technology continues to evolve, the role of reliable and accessible diagnostic tools like GaitKeeper will become increasingly significant. Gaitkeeper’s ability to integrate into various care models could advance proactive health management and facilitate the early detection of health issues, positioning gait speed measurements as a central component of patient care.

## Data Availability

The datasets generated during and/or analyzed during the current study are not publicly available due to privacy considerations and the potential risk of participant identification. Requests for further information about the datasets can be directed to the corresponding author.
